# Artificial intelligence–derived coronary plaque features predict incident acute ischaemic stroke independent of cardioembolic risk factors

**DOI:** 10.1093/ehjimp/qyaf069

**Published:** 2025-05-26

**Authors:** Robert Luceri, Francesca Calicchio, Fatima Amin, Livia De Sousa Domingues Da Silva, Eric Hu, Najah Khan, Gavin Cobb, Elizabeth Epstein, Melody Hermel, Shawn Newlander, Mary Kalafut, Austin Robinson, Jorge Gonzalez, George Wesbey

**Affiliations:** Department of Medicine, Scripps Clinic, 10666 N Torrey Pines Rd, La Jolla, CA 92037, USA; Department of Cardiology, Scripps Prebys Cardiovascular Center, Scripps Memorial Hospital, 9850, Genesee Ave, La Jolla, CA 92037, USA; Charles R. Drew University of Medicine and Science, Los Angeles, CA 90059, USA; Department of Cardiology, Scripps Prebys Cardiovascular Center, Scripps Memorial Hospital, 9850, Genesee Ave, La Jolla, CA 92037, USA; Scripps Health, La Jolla, CA 92037, USA; Department of Cardiology, Scripps Prebys Cardiovascular Center, Scripps Memorial Hospital, 9850, Genesee Ave, La Jolla, CA 92037, USA; Department of Medicine, Scripps Clinic, 10666 N Torrey Pines Rd, La Jolla, CA 92037, USA; Department of Cardiology, Scripps Prebys Cardiovascular Center, Scripps Memorial Hospital, 9850, Genesee Ave, La Jolla, CA 92037, USA; Department of Cardiology, Scripps Health & United Medical Doctors, La Jolla 92037, USA; Scripps Health, La Jolla, CA 92037, USA; Department of Medicine, Scripps Clinic, 10666 N Torrey Pines Rd, La Jolla, CA 92037, USA; Department of Cardiology, Scripps Prebys Cardiovascular Center, Scripps Memorial Hospital, 9850, Genesee Ave, La Jolla, CA 92037, USA; Department of Cardiology, Scripps Prebys Cardiovascular Center, Scripps Memorial Hospital, 9850, Genesee Ave, La Jolla, CA 92037, USA; Department of Cardiology, Scripps Prebys Cardiovascular Center, Scripps Memorial Hospital, 9850, Genesee Ave, La Jolla, CA 92037, USA

## Introduction

Ischaemic stroke, a leading cause of morbidity and mortality worldwide, results from an interruption in blood flow causing inadequate cerebral perfusion. The most common aetiology is embolic disease, which has multiple mechanisms. Coronary computed tomography (CT) imaging has been used to assess stroke risk by analysing mitral annular calcification (MAC), coronary artery calcification, and left atrial volume index (LAVI).^1,2,3^ Artificial intelligence (AI)–derived coronary CT angiography (CCTA) atherosclerotic plaque characteristics have been shown to predict atrial fibrillation (AF),^4^ but their role in predicting acute ischaemic stroke (AIS) has not been investigated. This study examines the association between CCTA-derived plaque features and AIS after adjusting for cardioembolic risk factors.

## Methods

This was a single-centre retrospective case-control study. The Scripps Epic database was queried from 1 April 2017 through 31 December 2024 for all adults with prior CCTA. AIS was identified by electronic medical record clinical documentation and confirmed by neuroimaging. Patients with stroke prior to CCTA, haemorrhagic stroke, trauma, periprocedural stroke, thrombus or vegetation on imaging, cardiac bypass surgery, cardiac stents, or cardiac valve procedures were excluded. Controls were identified as patients without subsequent development of stroke after CCTA with the same exclusion criteria as the study cohort. Controls were selected 1:1 by nearest neighbour propensity matching based on age, sex, smoker status, hypertension, hyperlipidaemia, and diabetes. Propensity matching was conducted using MatchIt (v4.5.2) in R (v4.0.3).

Anonymized DICOM CCTA images were analysed by CLEERLY laboratory using US Food and Drug Administration approved machine learning to quantify vessel, lumen, and compositional plaque volumes. The lab was blinded to AIS status. Specific coronary atherosclerotic plaque features that were analysed included calcified plaque (CP) volume, non-CP (NCP) volume, total plaque volume (TPV), per cent atheroma volume (PAV), and PAV of CP. LAVI was calculated using the Total Segmentator plugin to 3D Slicer, as previously validated.^4^ MAC presence was verified by visual inspection of pre-contrast gated CT. AF was diagnosed at any point in the patient’s history. Plaque characteristics were compared using Mann–Whitney test. Multivariate Cox proportional hazard ratio (HR) analysis was performed in R with the survival package while adjusting for the covariates MAC, AF, and LAVI. Data analysis was performed in Python (v3.7.3) using Jupyter Notebook and Anaconda (v23.1.0).

## Results

**Figure 1:**
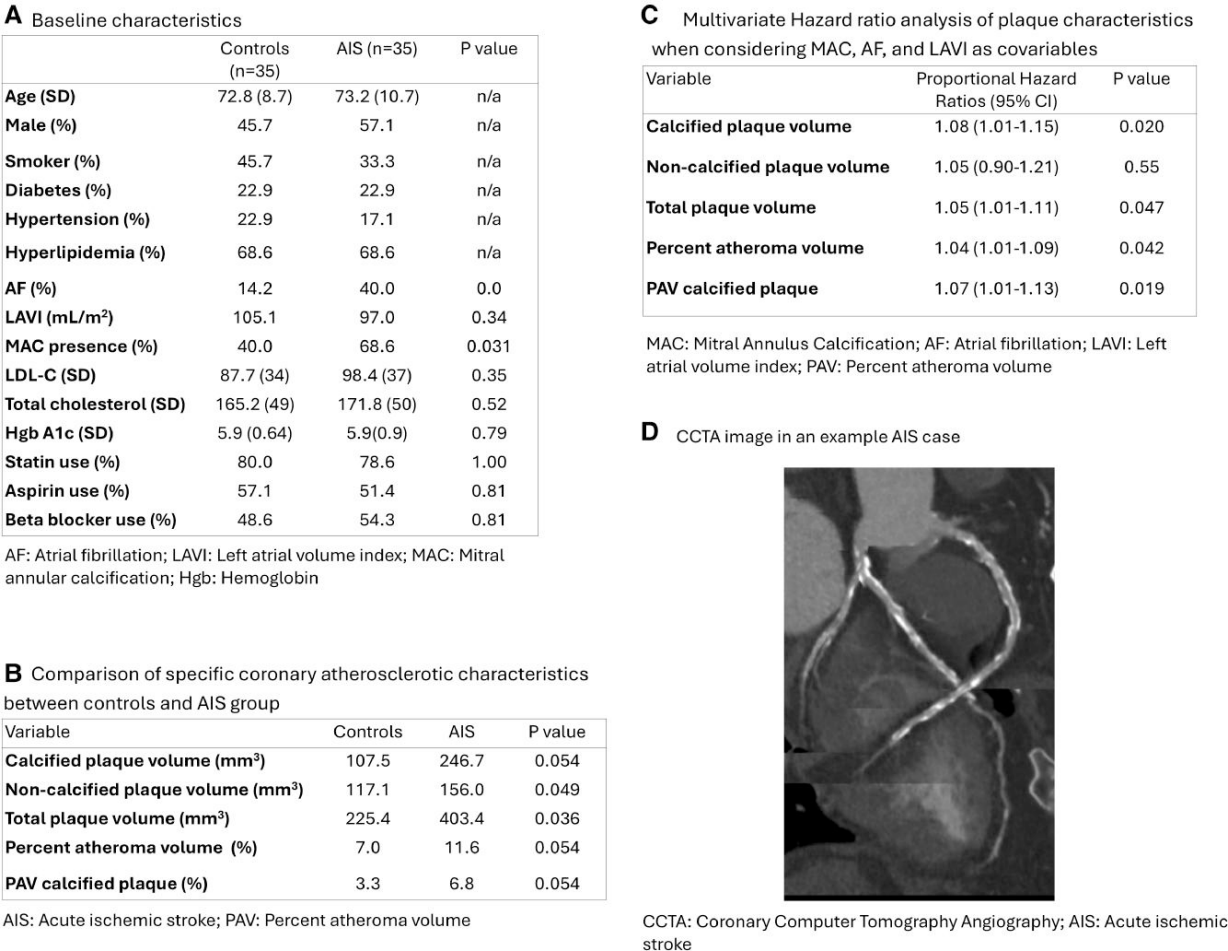
Baseline characteristics of the study population and analysis of AI-derived coronary plaque characteristics comparing the acute ischaemic stroke and control group.

There were 35 patients and 35 controls. Mean time to stroke was 854 days. The baseline characteristics of the propensity matched study population are shown in *Figure 1A*. There was a higher incidence of AF (*P* = 0.032) and MAC (*P* = 0.031) in the stroke group, but there was no significant difference in LAVI (*P* = 0.34). In terms of plaque characteristics, NCP volume (stroke 156 mm^3^ vs. control 117mm^3^, *P* = 0.049) and TPV (stroke 403 mm^3^ vs. control 225 mm^3^, *P* = 0.036) were significantly higher in the stroke group (*Figure 1B*). While accounting for the difference in AF, LAVI, and MAC between the study groups, multivariate HR analysis revealed a significantly increased risk of AIS associated with total CP (HR 1.08, *P* = 0.020), TPV (HR 1.05, *P* = 0.047), PAV (HR 1.04, *P* = 0.042), and PAV of CP (HR 1.07, *P* = 0.019) (*Figure 1C*).

## Discussion

To our knowledge, this is the first study to use AI-derived analysis of CCTA to identify coronary plaque features linked to the development of AIS. Patients with AIS had significantly higher NCP volume and TPV. When adjusting for MAC, AF, and LAVI, the total CP volume, TPV, and PAV of CP were associated with a significantly increased risk of AIS. This suggests that AI-derived coronary plaque characteristics may independently predict both cardiovascular events^4^ and neurovascular events, strengthening AI-derived CCTA plaque analysis as an atherosclerotic cardiovascular disease risk stratification tool.

## Data Availability

Data will be available to share with interested investigators through reasonable request with the study PIs, following appropriate data sharing agreements consistent with institutional policies and complying with IRB standards.

## Lead author biography

Dr Robert Luceri is a current resident physician at Scripps Clinic in San Diego, CA, USA. Dr Luceri earned his Bachelor of Science degree at the University of Notre Dame and then earned his Doctor of Medicine at Rutgers New Jersey Medical School. He served in the US Navy as a Medical Officer prior to commencing post-graduate training in internal medicine residency at Scripps Clinic. He plans to pursue a career in cardiology and is interested in preventive cardiology and cardiac imaging.
